# Evolution, Clinical and Microbiological Characteristics of Invasive Pneumococcal Disease since the Introduction of the Pneumococcal Conjugate Vaccine 13-Valent in Adults over 18 Years Old

**DOI:** 10.3390/vaccines9020093

**Published:** 2021-01-27

**Authors:** Juan Buades, Ines Losada, Juan González-Moreno, Maria Peñaranda, Laia Vilaplana, Nuria Roda, Adelaida Rey, Adrian Rodriguez, Margarita Garau, Enrique Ruiz de Gopegui, Antoni Serra, Juan Saurina, Antoni Payeras

**Affiliations:** 1 Internal Medicine Service, Son Llàtzer University Hospital (HUSLL), 07198 Palma, Spain; ialosada@hsll.es (I.L.); jgonzalez4@hsll.es (J.G.-M.); adrian.rodriguez@hsll.es (A.R.); apayeras@hsll.es (A.P.); 2 Institute of Health Research of the Balearic Islands (IdISBa), 07120 Palma, Spain; 3 Infectious Diseases Section, Hospital Universitario Son Espases (HUSE), 07120 Palma, Spain; maria.penaranda@ssib.es; 4 Department of Internal Medicine, Hospital de Manacor, 07500 Manacor, Spain; lvilaplana@hmanacor.org (L.V.); nroda@hmanacor.org (N.R.); 5 Internal Medicine Service, Hospital de Inca, 07300 Inca, Spain; adelaida.rey@hcin.es; 6 Microbiology Service, Son Llàtzer University Hospital (HUSLL), 07198 Palma, Spain; margagaraucolom2@gmail.com; 7 Microbiology Service, Hospital Universitario Son Espases (HUSE), 07120 Palma, Spain; enrique.ruiz@ssib.es; 8 Microbiology Service, Hospital de Manacor, 07500 Manacor, Spain; tserra@hmanacor.org; 9 Microbiology Service, Hospital de Inca, 07300 Inca, Spain; juan.saurina@hcin.es

**Keywords:** pneumococcal disease, invasive pneumococcal disease, pneumococcal conjugate vaccine, serotypes, antibiotic sensitivity

## Abstract

Invasive pneumococcal disease (IPD) presents high mortality in the population at risk. The aim of this work is to know the evolution, clinical and microbiological characteristics of IPD in the adult population of Majorca, since the introduction of a public funded program for pneumococcal conjugate vaccine (PCV-13) in the pediatric population in the Balearic Islands in 2016. For this purpose, a retrospective multicenter study was carried out in which all episodes of IPD in adult patients from the four hospitals of the public health system of Majorca were included, comparing the periods between 2012 and 2015 and between 2016 and 2019. Clinical variables, serotypes and antibiotic sensitivity were collected. There were 498 cases of IPD; 56.8% were male with a mean age of 67 (standard deviation: 16). Most infections were bacterial pneumonias (73.7%). Of the total cases, 264 (53%) presented complications. Of the 498 cases, 351 strains were obtained, of which 145 (41.3%) belong to vaccinal serotypes (included in the PCV-13 vaccine) and 206 (58.7%) to non-vaccinal serotypes (not included in the PCV-13 vaccine). The percentage of IPD caused by vaccinal serotypes was lower in the second period (47.8% vs. 34.5%; *p* = 0.012).

## 1. Introduction

*Streptococcus pneumoniae (S. pneumoniae)* is the most common microorganism causing community-acquired pneumonia and has a high burden of morbidity and mortality worldwide [1,2]. Invasive pneumococcal disease (IPD), in its different forms of presentation (bacterial pneumonia, septicemia or meningitis), presents high morbidity and mortality. This is especially evident in at-risk populations, such as those under 2 and over 65 years of age, as well as patients with immunodeficiencies of any type and those with anatomical or functional asplenia [3,4]. Moreover, IPD is also observed in immunocompetent persons with certain comorbidities, such as cardiovascular, respiratory, hepatic, renal and metabolic diseases [1,2,3,4]. There are up to 100 different serotypes currently described, some more associated with IPD and with resistance to different antimicrobials [5,6] Although vaccines against pneumococcus have been available for some time, it has been in recent decades, with the introduction of pneumococcal conjugate vaccine (PCV), that significant progress has been made in preventing these infections [7,8].

In 2001 in Spain, the 7-valent conjugate vaccine (PCV-7) containing capsular polysaccharides of the *S. pneumoniae* serotypes 4, 6B, 9V, 14, 18C, 19F and 23F was included in childhood immunization programs and later in 2010, after the authorization of PCV-13, coverage was extended to some emerging serotypes, such as 1, 3, 5, 6A, 7F and 19A, responsible for more serious infections or those that posed resistance problems [5,6,9]. Where childhood vaccination programs have been implemented, reductions in the incidence of IPD in both children and adults have been observed with PCV [10,11]. In addition, different scientific societies recommend vaccination with PCV for immunodeficient adult patients, those with comorbidities or those over 65 years of age [12,13].

In the Balearic Autonomous Community, public funding for vaccination with PCV in the pediatric population was approved in mid-2016 [14], and until then, it had only been recommended by pediatricians, and the child’s parents had to pay for it. Vaccination of adult patients is not currently funded by the public health system in our setting, so there is great variability when it comes to implementing the recommendations of the different scientific societies, which depends on criteria as different as the patient’s characteristics, the physician’s awareness or the environment, whether hospital or outpatient, and where the care is provided [15]. The Spanish Ministry of Health has recommended vaccination with Pneumococcal Polysaccharide Vaccine (PPSV23) since 2004 to adults aged 65 and older [16], and a more recent recommendation is the same. In our community, these recommendations apply [17].

The aim of this study is to know the evolution, clinical and microbiological characteristics of IPD in the adult population of Majorca before and after the financing of PCV in a pediatric population. For this purpose, two periods between 2012 and 2015 and between 2016 and 2019 are compared. In addition, complications in that population and the profile of antibiotic sensitivity to the different serotypes most prevalent since the authorization of the PCV-13 in Spain and more specifically in our community were analyzed. In this way, it is intended to explore the opportunities of improvement in order to prevent these infections with the introduction of future vaccines with a higher number of conjugated serotypes.

## 2. Material and Methods

A retrospective multicenter study of all the cases of IPD assisted in the 4 public hospitals of Majorca (Hospital Universitario Son Espases, Hospital Universitario Son Llàtzer, Hospital Comarcal de Manacor and Hospital Comarcal de Inca) in the period from 2012 to 2019 was carried out. These 4 centers serve a total population of 896,014 inhabitants (2019 census) [18].

All episodes of IPD in patients over 18 years of age treated consecutively in any of the 4 participating hospitals were included. IPD was defined as isolation of S. pneumoniae in any sterile fluid (blood, pleural fluid, cerebrospinal fluid, ascitic fluid, or joint fluid) in patients with clinical symptoms or signs of infection. These data are transferred monthly from the microbiology department and transferred to a common database of the 4 hospitals participating in the study, taking into account the same definition previously described.

In all episodes of IPD, the following variables were collected: age, sex, origin of infection (community, nosocomial or care-associated), clinical diagnosis, toxic habits, previous antibiotic treatment, vaccination against pneumococcus or influenza, comorbidities, FINE severity index for pneumonias, complications, exitus and microbiological characteristics such as *S. pneumoniae* serotype and antibiogram.

The antecedent of anti-pneumococcal or flu vaccination was collected through the review of the patient’s hospital medical history and after consulting the vaccine registry of the *“Història de Salut”* medical records site of the Servei Balear de Salut [19].

The biological samples were processed in the microbiology services of each of the participating centers following the usual laboratory procedures [20]. The sensitivity study to the different antibiotics was carried out by E-test^®^ (bioMérieux, Marcy l’Etoile, France) for penicillin and cefotaxime and disc-plate (Oxoid^®^ Basingstoke, or Neo-Sensitabs™, Taastrup, Denmark) for ampicillin, erythromycin, tetracyclines, cotrimoxazole, levofloxacin and vancomycin, following the recommendations and cut-off points established by CLSI (Clinical Laboratory Standards Institute) during the first period, and EUCAST (European Committee on Antimicrobial Testing) since January 2016. To carry out the serotyping, the strains were sent to the Pneumococcal Reference Laboratory, Centro Nacional de Microbiología, Instituto Carlos III (Madrid, Spain).

A descriptive analysis of the clinical and microbiological characteristics of all the episodes was carried out. Then, the annual incidence of IPD was calculated using the population data of Majorca, according to the census of the Institut d’Estadística de les Illes Balears IBESTAT [18]. Finally, a bivariate analysis was carried out to compare prognostic variables, complications and microbiological characteristics during two equal time periods: 2012–2015 and 2016–2019. For the comparison of the qualitative variables a Chi-square test was used, applying Fisher’s correction in those cases where it was indicated, and for the quantitative variables, the Student’s *t*-test was used.

The data were included in an anonymized database, using the SPSS (IBM, Armonk, NY, USA) statistical package version 22.0.

Informed consent was obtained from the patients or their legal representatives in case of disability or death and the study was approved by the Research Commission of each of the participating centers.

## 3. Results

During the study period, 498 IPD cases affecting adults aged 18 and older were included, of which 283 (56.8%) were male, with a mean age of 67 (SD: 16). Most, 478 (96%), required hospitalization and the origin of the infection was in 384 (77.1%) community acquired, 97 (19.5%) healthcare-associated and 17 (3.4%) nosocomial.

Regarding diagnosis, 367 (73.7%) were bacterial pneumonias, 68 (13.2%) bacteremias, 47 (9.4%) meningitis, 10 (2%) peritonitis and other causes (arthritis and empyema) with marginal incidence.

**Figure 1:**
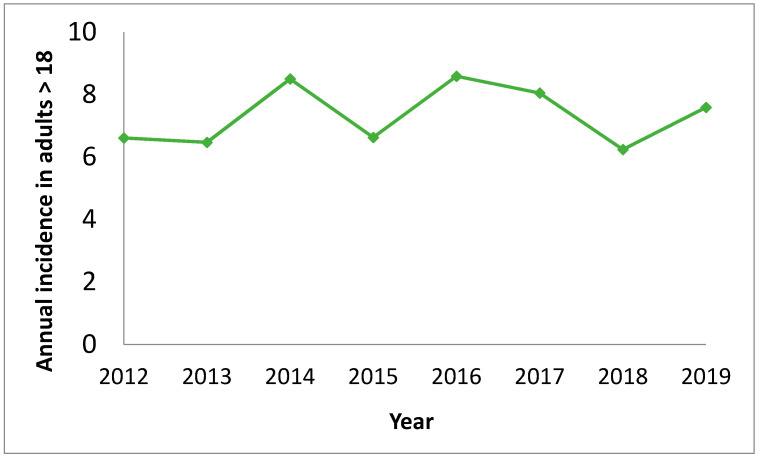
Annual incidence of invasive pneumococcal disease in adults aged 18 and older (cases per 100,000 population).

The main comorbidities presented by the patients were: 158 (31.7%) chronic respiratory disease, 117 (23.5%) diabetes, 107 (21.5%) cancer, 101 (20.3%) heart failure, 61 (12.2%) liver disease, 37 (7.4%) hematological disease, 26 (5.2%) chronic renal failure, 21 (4.2%) connective tissue diseases and 15 (3%) AIDS. The median (P25–P75) of Charlson’s index was 2 (1–4). The annual incidence, only including the adult population, was 6.64 × 10000 inhabitants, and did not suffer significant variations during the years studied (Figure 1). In addition, there have been no differences in the baseline characteristics of the population studied between periods.

Fine’s index for pneumonia was 109 (standard deviation 39.7). Complications occurred in 264 (53%) cases: 147 (29.5%) sepsis, 87 (17.5%) septic shock, 58 (11.6%) pleural effusion, 15 (3%) empyema, 13 (2.6%) distant metastasis and 2 (0.4%) endocarditis. 69 (13.9%) of the cases required oral endotracheal intubation (ETI) and 42 (8.4%) non-invasive ventilation. Overall, 101 (20.3%) cases were admitted to ICU and 91 (18.3%) patients died, 59 (11.8%) in relation to the infection. For definition of sepsis and sepsis shock we follow the new consensus definition “defined as life-threatening organ dysfunction caused by a dysregulated host response to infection” and “Septic shock is a subset of sepsis in which underlying circulatory and cellular/metabolic abnormalities are profound enough to substantially increase mortality” [21].

Analyzing the overall vaccination rate in adults during the study period, only 41 cases (8.2%) had received vaccination against pneumococcus 22 (53%) during the first period and 19 (47%) during the second period. In addition, 70 cases (14.4%) had received vaccination against influenza. No differences were shown with regard to the evolution between the periods under study as shown below in Table 1.

**Figure 2:**
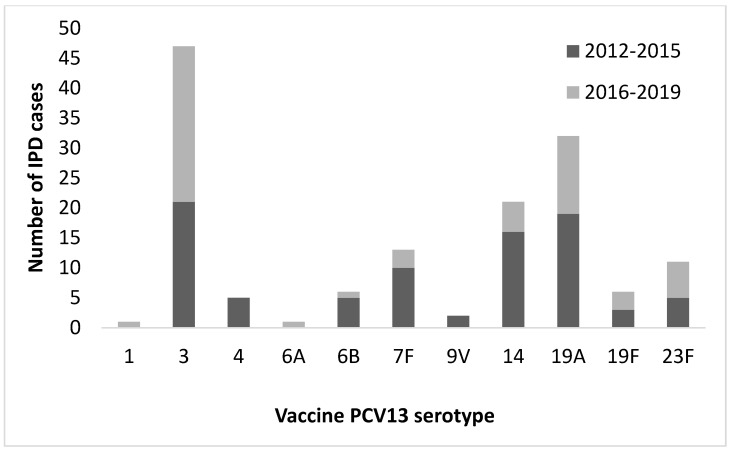
Distribution of pneumococcal conjugate vaccine PCV-13 serotypes in adults over 18 years of age (*n* = 145) throughout the study period (2012–2019).

**Figure 3:**
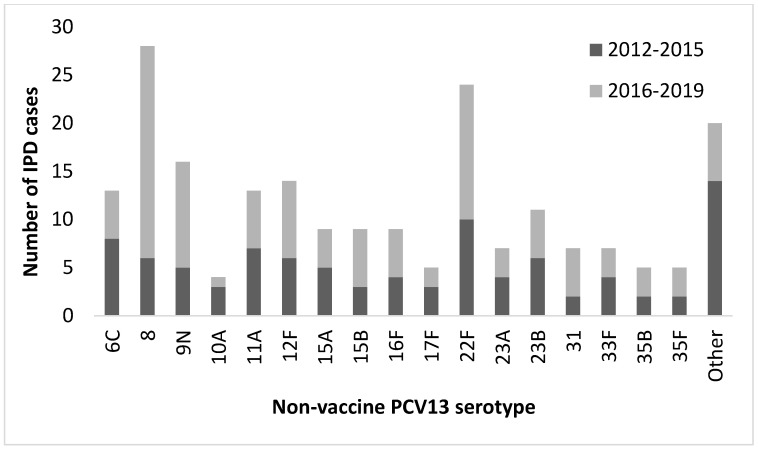
Distribution of non-vaccine PCV-13 serotypes (*n* = 206) in adults over 18 years of age throughout the study period (2012–2015 and 2016–2019). Other serotypes (N): 10B (1), 11C (1), 13 (1), 15C (1), 17A (1), 20 (2), 22 (1), 22A (1), 24F (3), 28 (1), 29 (1), 34 (2), 38 (2), 40F (1), 7C (1).

Of the 498 cases, 351 strains were obtained, of which 145 (41.3%) belonged to vaccinal serotypes (included in the PCV-13 vaccine) and 206 (58.7%) to non-vaccinal serotypes (not included in the PCV-13 vaccine). It should be noted that 122 of those 206 serotypes were included in the PPV23. Their distribution is shown in Figure 2 and Figure 3, respectively.

The distribution of the most prevalent diagnoses of the serotyped strains according to whether they were included in the PCV-13 is shown in Table 2.

When comparing the periods 2012–2015 and 2016–2019, a significant decrease in infections by vaccine serotypes was observed (47.8% vs. 34.5%; *p* = 0.012), as shown in Table 3.

**Figure 4:**
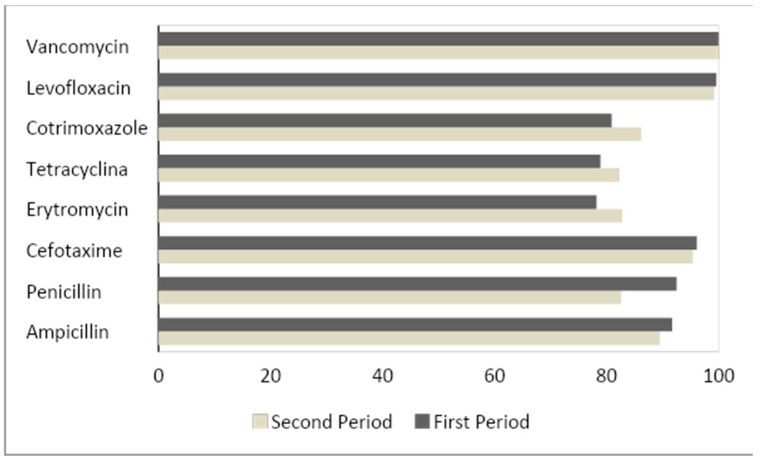
Antibiotic sensitivity profile of our population distributed by periods of study. The results are expressed as a % of sensitivity to each of the different antibiotics. Period 1 (2012–2015); Period 2 (2016–2019).

The percentages of antibiotic sensitivity by periods are shown in Table 3 where significant differences (*p* < 0.001) were only observed in a higher sensitivity to penicillin in the first period. These differences are due to the large difference between the sensitivity cut-off points for penicillin in non-meningitis cases proposed by the CLSI, followed during the first period of the study (s ≤ 2 µg/mL) compared with the EUCAST cut-off points applied since 2016 (s ≤ 0.06 µg/mL). The rest of the antibiotics presented a similar sensitivity profile in both periods as shown in Figure 4.

## 4. Discussion

In this retrospective study of the evolution of invasive pneumococcal disease for the last 8 years, a significant decrease in the percentage of vaccine serotypes between the two periods has been observed. In the sample, representative of our community, the most prevalent diagnosis was bacterial pneumonia followed by bacteremia. The comorbidities have not shown differences in the different periods, as well as the annual incidence has remained stable during the whole studied period. There were also no differences in terms of complications, related mortality or antibiotic sensitivity in the periods studied.

The study includes a representative population sample, since it includes the four hospitals of the public network of Majorca. The profile of the population, divided in two periods, before and after the authorization of the PCV-13 in the pediatric population, does not show differences in its basal characteristics and the incidence of cases remains stable. The low vaccination rate is noteworthy, taking into account that these data are taken from the public network, both inpatient and outpatient [19]. The possible explanations for this fact are the absence of an established adult pneumococcal vaccination program and the fact that during the years prior to the inclusion of childhood vaccination programs, it was privately funded and therefore the vaccination rate is underestimated.

In general, the overall rate of IPD has remained stable during all the years included in this study despite the differences in serotypes that are detailed below. As for the severity and etiology of infections, no significant differences are shown between periods. Recent studies show the impact of PCV-13 on pneumococcal infection in patients with COPD, demonstrating an overall decrease in PCV-13 serotypes in that population [22]; this effect may not be remarkable in our series due to lack of sample size.

Based on our data, we see that there is a potential trend to decrease the rate of pneumococcal infections if the guidelines are applied early as already observed in the CAPITA study [13]. This argument is reinforced by studies in our country that have developed a simulated economic model to evaluate the cost of the vaccination strategy in the population over 65 years of age, showing itself to be an efficient strategy with improved quality of life and life expectancy [23,24]. It is remarkable to highlight the low vaccination rate in the high-risk population in our environment, in line with the country’s data. More and more studies are emerging with evidence in cost-effective terms in favor of health interventions that implement vaccination in the adult population [24].

A significant decrease has been observed in the incidence of vaccinal serotypes, that inversely leads to an increase in incidence by non-vaccinal serotypes. One of the possible reasons, despite the low rate of vaccinated adult patients, could be the herd effect that implies vaccination in pediatric age (decreasing the carriers in this population) and its indirect effect on the non-vaccinated population (especially in elderly population) where it is known that the incidence of IPD is higher. In turn, this indirect effect is responsible for the increase in non-vaccinated serotypes, known as serotype replacement [25]. As it already happened in multicenter studies carried out in our country and at a European level, a significant reduction in IPD caused by serotypes included in PCV-13 has been seen. It should be noted that this effect has been magnified in communities that had included the universal childhood vaccination program earlier [26]. Another reason could be cross immunity as observed against serotypes not included in PCV-10 before the implementation of PCV-13, with a decrease in the incidence of serotype 19A (which is not included in PCV-10) [27,28]. In this way, our study coincides with series published in our region and in Europe, where a decrease in infections caused by vaccine serotypes in adults has been observed [25,29]. Therefore, we believe that an indirect benefit of vaccination with PCV-13 is possible, which at the moment is not completely apparent due to the low rate of vaccination, especially in adults, with a greater temporal perspective being necessary to be able to consolidate this benefit and to be able to observe a decrease in global incidence of IPD.

In spite of the progressive change of serotypes, a decrease in the global incidence of IPD has not been observed. Recent publications attribute this fact to several factors, such as the adult population’s immunological status, which may not fully reflect the vaccine’s immunogenic potential, but they still stress that clinical trials are necessary to obtain more valid results and to be able to assess the vaccine’s efficacy [30]. As already observed in the replacement of PCV-7 by PCV-13, the debate on how it affects the incidence of disease caused by non-vaccine serotypes has created controversy, particularly in the adult population. A possible explanation for this fact and for marked differences between countries could be due to differences in sampling (hospital or primary care center), risk factors in different populations, strategies in the vaccination schedule and the interaction between serotypes and molecular epidemiology. This would explain the differences between the European and American series [31]. Despite this, no increase in the severity of infections or mortality has been observed, nor in the percentage of antimicrobial resistance, despite the change in serotypes, unlike what occurred with the introduction of PCV-7 [32].

Secondary studies have observed an increase in the incidence of non-vaccinated serotypes such as 8 that would currently be included in the new 15- and 20-serotype conjugate vaccines [33]; however, as is the case in the child population, the rate of IPD caused by serotype 3 (included in PCV-13) has not been reduced in adults [32,34]. In our study, a higher incidence of serotypes 8 and 22F is observed, which, as previously mentioned, would be included in the new conjugated vaccines still in the experimental phase. It should be noted that there were 31 cases of IPD for non-vaccinal serotypes that would be included in the VCN-15 (serotypes 22F and 33F) and 99 cases that could have been covered by the new PCV-20 vaccine (which includes 22F, 33F, 8, 10A, 11A, 12F, 15B), both vaccines still in the experimental phase, which could open new hope for the reduction in the overall rate of IPD.

As limitations of our study, it should be noted that it is a retrospective study, that only patients from the public health network are included, and therefore the vaccination data may have been underestimated because they were collected from the clinical history or the health history [15]. Due to the data collection system, it is not possible to determine exactly how many of them received PPV23 or PCV-13 and if some received a combination of both. Thus, the strengths of the study are that it covers the entire network of hospitals of the Public Health System of Majorca for many years, with a follow-up by a team with a previous scientific background [29,35,36] and that it is a study with a significant number of cases of IPD that are representative of the population of the entire island of Majorca.

## 5. Conclusions

We observed a decrease in the incidence of IPD by vaccine serotypes since the inclusion of VCN-13 in the vaccination calendar. There is a maintained stability in the global rate of IPD which requires further monitoring to assess current vaccination strategies in the future. There are a total of 130 cases of NID by non-vaccinal serotypes that would be covered by the new VCN-15 and VCN-20 conjugate vaccines.

## Figures and Tables

**Table 1:** Comparison of complications in patients with invasive pneumococcal disease between period 1 (2012–2015) and period 2 (2016–2019). The results are expressed as N (%).

Complications	Period 1 *n* (%)	Period 2 *n* (%)	Significance
Pleural effusion	27 (11.5%)	31 (11.9%)	NS
Empyema	8 (3.4%)	7 (2.7%)	NS
Metastasis	3 (1.3%)	10 (3.8%)	NS
Endocarditis	0 (0%)	2 (0.8%)	NS
Severe sepsis	68 (28.9%)	79 (33.8%)	NS
Septic shock	42 (17.9%)	45 (17.3%)	NS
ICU	45 (19.1%)	56 (21.3%)	NS
Related Exitus	33 (14.1%)	26 (10%)	NS

Exact Fisher test or Chi-square test.

**Table 2:** Distribution of diagnoses according to serotypes included or not in the PCV-13 vaccine.

Diagnosis	Vaccine Serotypes *n* (%)	Non-Vaccinal Serotypes *n* (%)	Significance
Bacterial Pneumonia	107 (42.3%)	146 (57.7%)	NS
Bacteremia	15 (32%)	32 (68%)	NS
Meningitis	17 (42.5%)	23 (57.5%)	NS
Peritonitis	3 (42.9%)	4 (57.1%)	NS

**Table 3:** Comparative % of IPD from PCV-13 serotypes during the periods of the study.

	2012–2015	2016–2019	*p*-Value
Infection by PCV-13 vaccine serotype (%)	47.8	34.5	0.012

## Data Availability

The data is available under reasonable request to the corresponding author.
